# Elevated hydrostatic pressure disturbs expression of growth factors in human renal epithelial cells

**DOI:** 10.1371/journal.pone.0310001

**Published:** 2024-09-06

**Authors:** Chen Yan, Jie Xiao, Yong-Hua Peng, Tao-Sheng Li

**Affiliations:** 1 Department of Stem Cell Biology, Atomic Bomb Disease Institute, Nagasaki University, Nagasaki, Japan; 2 Department of Nephrology, The First Affiliated Hospital of Guangzhou Medical University, Guangzhou, P.R. China; Tohoku University School of Medicine: Tohoku Daigaku Daigakuin Igakukei Kenkyuka Igakubu, JAPAN

## Abstract

Obstructive uropathy is a common kidney disease caused by elevated hydrostatic pressure (HP), but relevant molecular and cellular mechanisms have not yet been well understood. In this study, we *ex vivo* investigated the effects of elevated HP on human renal epithelial cells (HREpCs). Primary HREpCs were subjected to 100 cmH_2_O HP for 8 or 48 h. Then, the cells were cultured without HP stimulation for another 24 h or 72 h. Cell morphology showed almost no change after 8h HP treatment, but exhibited reversible elongation after 48h HP treatment. HP treatment for 8 h increased the expression of *TGFB1* and *VEGFA* but decreased the expression of *CSF2* and *TGFB2*. On the other hand, HP treatment for 48 h downregulated the expression of *CSF2*, *TGFB2*, *PDGFB*, *VEGFA*, and *VEGFB*, while upregulated the expression of *TGFB3*. Interestingly, all changes induced by 48 h HP treatment were detected more severe compared to 8 h HP treatment. In conclusion, elongated *ex vivo* HP loading to renal epithelial cells induces reversible changes on cell morphology and disturbs the expression of several growth factors, which provides novel mechanistic insight on elevated HP-caused kidney injury such as obstructive uropathy.

## Introduction

The kidneys are pivotal to maintaining the fluid and electrolyte balance. Obstructive uropathy is most commonly caused by malignancy, urolithiasis, and others [1]. Acute kidney injury (AKI) was present in over 70% of the patients with obstructive uropathy [1]. In obstructed human kidneys, the intrarenal pressures (IRPs) increase from a few cmH_2_O to more than 90 cmH_2_O [2]. Animal studies have proved that elevated IRPs can damage kidneys [3–5]. In a porcine study, hydrostatic pressure (HP) induces renal cellular injury within a few hours at IRPs of 20 cmH_2_O or greater [3]. Histological analysis also shows that elevated HP leads to marked vacuolization and degeneration in renal tubules [4, 5]. Additionally, the release of the ureteral obstruction leads to either complete or partial reversal of kidney injury, contingent upon the severity and duration of the obstruction [6, 7].

The effects of HP on many cell types, including hepatic stellate cells, renal cells, bladder fibroblasts, and cancer cells, have been investigated [8–13]. These studies show that HP can increase cell proliferation, cell detachment, tissue fibrosis, epithelial-mesenchymal transition (EMT), and cancer metastasis [8–13]. However, the effects of HP on renal epithelial cells have rarely been investigated. One study has shown that *in vitro* exposure of renal cells to 60 cmH_2_O HP results in increased cell number and rearranged microfilament structure [11]. Using a unilateral ureteral obstruction mouse model and primary human renal epithelial cells (HREpCs), we also found that elevated HP can induce renal fibrosis and EMT of renal epithelial cells [12, 14].

It has been demonstrated that various growth factors maintain kidney physiological functions and are involved in the pathogenesis of kidney disease [15]. In this study, we investigated how elevated HP affected the expression of growth factors in the HREpCs, and further interested to know whether the elevated HP-induced changes are reversible.

## Materials and methods

### Cell culture

HREpCs were purchased from ScienCell Research Laboratories (#4120, ScienCell). Cells were maintained in an epithelial cell medium (#4101, ScienCell) and cultured at 37°C in a humidified atmosphere of 5% CO_2_ and 95% air.

### HP loading

We used a pneumatic pressurizing system (Strex. Inc) to mimic mechanical stimuli of kidney cells experienced by obstructive uropathy. Briefly, 5 × 10^5^ HREpCs were seeded in 6 cm diameter culture dishes. After an overnight incubation, the cells were placed inside the pressure chamber. Then, the chamber was placed inside a CO_2_ incubator and connected to the control box outside the incubator via vacuum tubing. Cells were loaded to 100 cmH_2_O sustained HP for 8 or 48 h. To ensure cell viability, a 30 s gas exchange occurs every 60 min by releasing pressure and re-pressurizing the chamber.

After 8h (short-term) or 48h (long-term) treatment with 100 cmH_2_O HP, we evaluated the morphological change and gene expression level in cells immediately (HP 8 h + without HP 0 h) and after culturing without HP stimulation for another 24 h (HP 8 h + without HP 24 h) or 72 h (HP 8 h + without HP 72 h).

### Real-time PCR

RNA was isolated from cells using a NucleoSpin RNA plus kit (#740984.50, Takara, Japan). RNA concentration was measured using a NanoDrop 2000 spectrophotometer (Thermo Fisher Scientific, USA), and 1 μg of RNA was used to generate cDNA by using the PrimeScript IV 1st strand cDNA synthesis mix kit (#6215B, Takara, Japan). The SYBR Green Real-time PCR Master mix was used per the manufacturer’s instructions (#QPS-201, TOYOBO, Japan). Real-time PCR amplification reactions were performed using the CFX96 touch Real-time PCR detection system (Bio-Rad, USA). The fold change of gene expression was calculated using the 2^-ΔΔCT^ method. The results represent the mean of three independent samples. Glyceraldehyde-3-phosphate dehydrogenase (*GAPDH*) was analyzed as a reference gene. Colony-stimulating factor 1 (*CSF1*), colony-stimulating factor 2 (*CSF2*), transforming growth factor-beta 1 (*TGFB1*), transforming growth factor-beta 2 (*TGFB2*), transforming growth factor-beta 3 (*TGFB3*), platelet-derived growth factor A (*PDGFA*), platelet-derived growth factor B (*PDGFB*), vascular endothelial growth factor A (*VEGFA*) and vascular endothelial growth factor B (*VEGFB*) were evaluated. Primer sequences are shown in the 5’ to 3’ direction (S1 Table).

### Cell morphological analysis

Cell morphology was observed under phase contrast microscopy. The aspect ratio was defined as the longitudinal cell axis (major axis) divided by the transverse cell axis (minor axis). The aspect ratio was calculated using Fiji software [16, 17]. Each data set consists of measurements of 100 cells.

### Cell viability assays

Cells were seeded in 96-well culture plates at a density of 1 × 10^4^ cells per well and cultured overnight. The cells were then treated with or without 100 cmH_2_O HP for 48 hours. After treatment, cell viability assays were performed using the Cell Proliferation Kit I (MTT) (#11465007001, Roche, Switzerland). Briefly, MTT was added and incubated for 4 hours. Then, the formation of formazan from MTT was stopped by adding solubilization solution, and the absorbance of formazan was measured at 570 nm using a microplate reader (iMark, Bio-Rad, USA). We used the optical density (OD) value of control cells as a normalization control (100%).

### Immunofluorescence

Immunofluorescence for filamentous actin (F-actin) was performed by Alexa Fluor Plus 555 Phalloidin (#A30106, Thermo Fisher Scientific, USA) staining. Briefly, cells were fixed with 4% paraformaldehyde (#163–20145, Wako, Japan) for 10 minutes at room temperature. After washing with phosphate buffered saline (PBS), cells were permeabilized in 0.1% Triton X-100 (#X100, Sigma-Aldrich, USA) in PBS for 15 minutes. After washing with PBS, cells were incubated with Alexa Fluor Plus 555 Phalloidin at room temperature for 30 minutes in the dark. After washing with PBS, cells were incubated with NucBlue Fixed Cell ReadyProbes Reagent (DAPI, #R37606, Thermo Fisher Scientific) for 15 minutes. The immunofluorescence in cells was examined on a fluorescence microscope (BZ-X810, Keyence, Japan).

### Statistical analysis

All data were presented as the mean ± SD. Two-tailed Student’s t-test was used to compare continuous variables between two groups for normally distributed variables. Data between groups for normally distributed variables were compared by a one-way analysis of variance (ANOVA) followed by Tukey’s test. Data between groups for non-normally distributed variables were compared by Kruskal–Wallis test followed by Dunn’s test. A *p*-value less than 0.05 was considered significant. Data were analyzed with GraphPad Prism 9 (GraphPad Software, USA).

## Results

### Short-term HP treatment induced mild changes in cell morphology and growth factor expression in HREpCs

**Fig 1:**
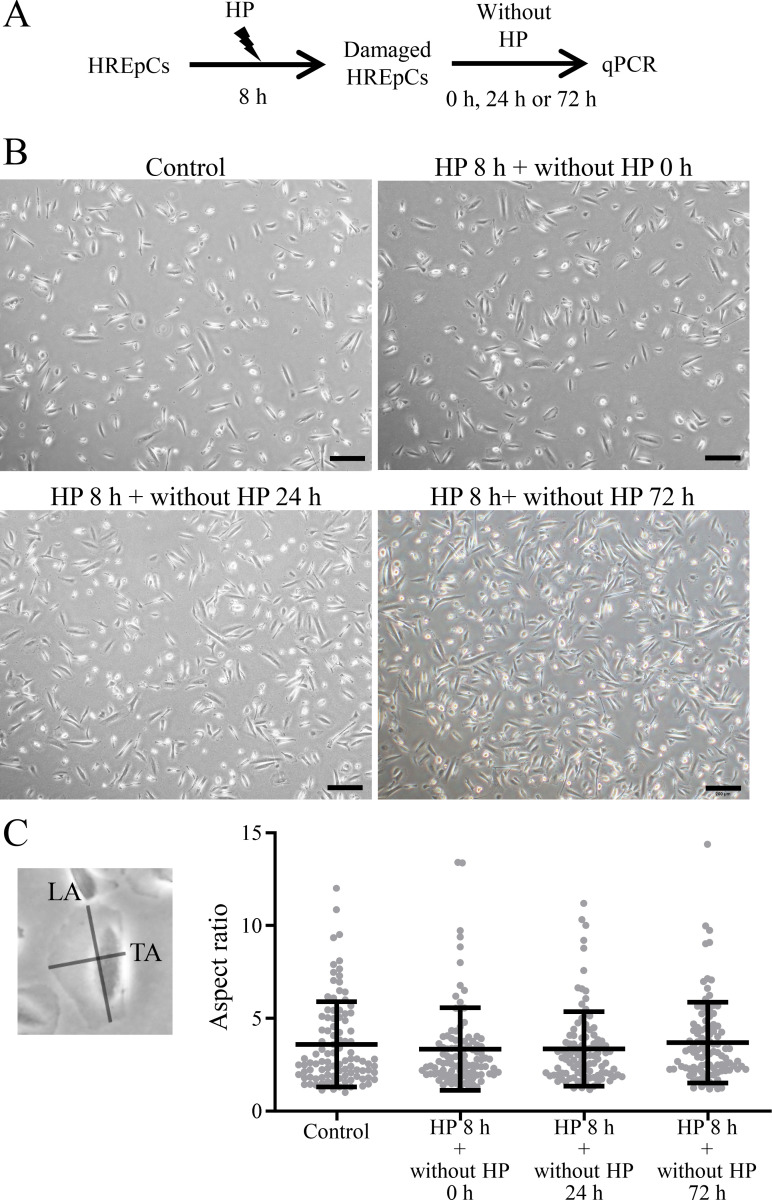
Morphological changes of HREpCs received 100 cmH_2_O HP treatment for 8 h. (A) Experimental protocol scheme. (B) Representative phase-contrast images show the morphology of cells (Scale bars: 200 μm). (C) Aspect ratio of cells. Each dot represents a single cell. *: *p* < 0.05 *vs*. control cells. LA: Longitudinal axis; TA: Transverse axis.

HREpCs were subjected to 100 cmH_2_O HP for 8 h, and then cultured without HP for another 24 h or 72 h (Fig 1A). Under phase contrast microscopy, we noticed that cell morphology and aspect ratio had almost no change induced by short-term (8 h) HP treatment (Fig 1B and 1C). The aspect ratio was defined as the longitudinal cell axis (major axis) divided by the transverse cell axis (minor axis) (Fig 1C).

**Fig 2:**
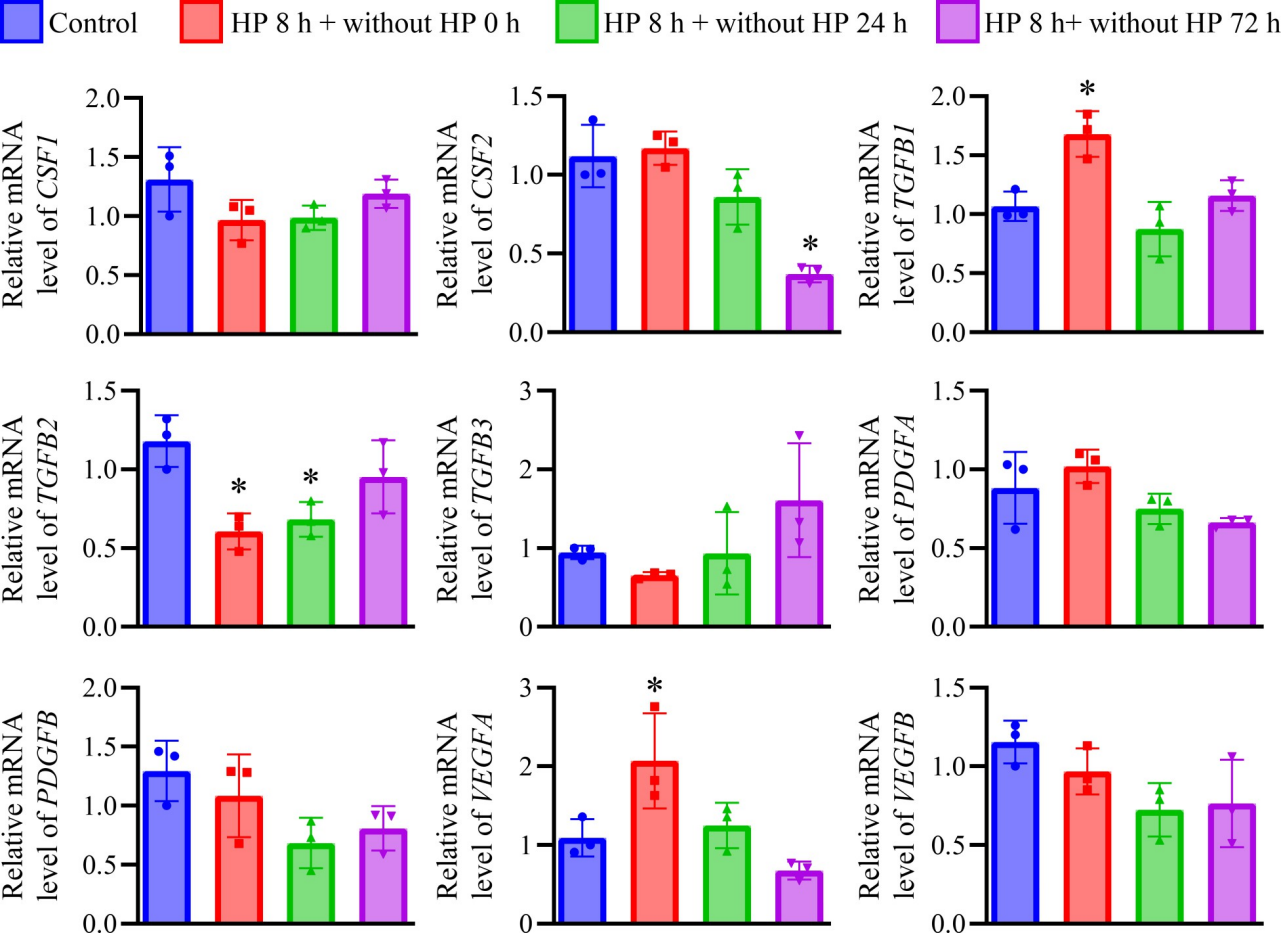
The expression of growth factors in HREpCs received 100 cmH_2_O HP treatment for 8 h. Quantitative analysis of the mRNA expression of *CSF1*, *CSF2*, *TGFB1*, *TGFB2*, *TGFB3*, *PDGFA*, *PDGFB*, *VEGFA*, and *VEGFB*. *: *p* < 0.05 *vs*. control cells.

We next investigated the expression of growth factors in HREpCs after short-term HP treatment. The expression of *CSF2* was not significantly changed by short-term HP treatment. However, the *CSF2* expression in the HP-treated cells decreased gradually with time and resulted in a significantly lower level at 72 h after removing from HP treatment (*p* < 0.05, *vs*. control, Fig 2). The expression of *TGFB1* and *VEGFA* were increased by short-term HP treatment (*p* < 0.05, *vs*. control, Fig 2), but returned to the basic level in 24h after removing from HP treatment. The expression of *TGFB2* was decreased by short-term HP treatment and returned to the basic level at 72h after removing from HP treatment (*p* < 0.05, *vs*. control, Fig 2).

### Long-term HP treatment induced severe changes in cell morphology and growth factor expression in HREpCs

**Fig 3:**
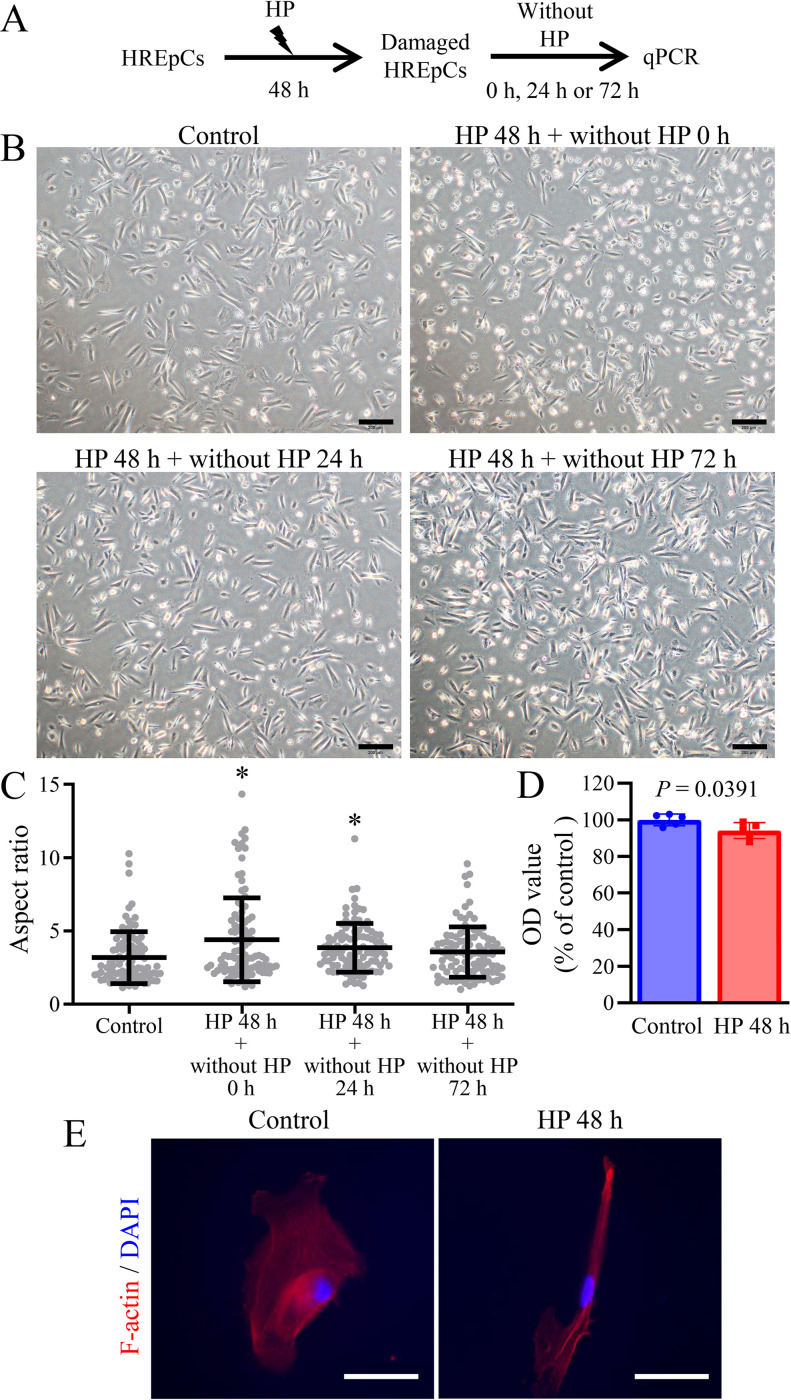
Morphological changes and cell viability of HREpCs received 100 cmH_2_O HP treatment for 48 h. (A) Experimental protocol scheme. (B) Representative phase-contrast images show the morphology of cells (Scale bars: 200 μm). (C) Aspect ratio of cells. Each dot represents a single cell. *: *p* < 0.05 *vs*. control cells. (D) Cell viability assays. (E) Immunofluorescence for F-actin by staining with Alexa Fluor Plus 555 Phalloidin (Scale bars: 50 μm).

We also investigated the long-term effect of HP treatment on HREpCs. The HP treatment period was extended to 48 h (Fig 3A). Under phase contrast microscopy, we observed some cells showing a round shape 48 h after HP treatment (Fig 3B). The cellular aspect ratio was increased by long-term (48 h) HP treatment, but returns to the basic level in 72 h after removing from HP treatment, suggesting a reversible elongation of the HP-treated cells (*p* < 0.05, *vs*. control, Fig 3C). An MTT assay indicated the cell viability was about 6% lower in HP-treated cells than control cells (*p* = 0.0391, Fig 3D), suggesting a slight but statistically significant effect on cell viability/metabolic activity. Staining cells with F-actin also showed that HP-treated cells presented elongated morphology compared to control cells (Fig 3E).

**Fig 4:**
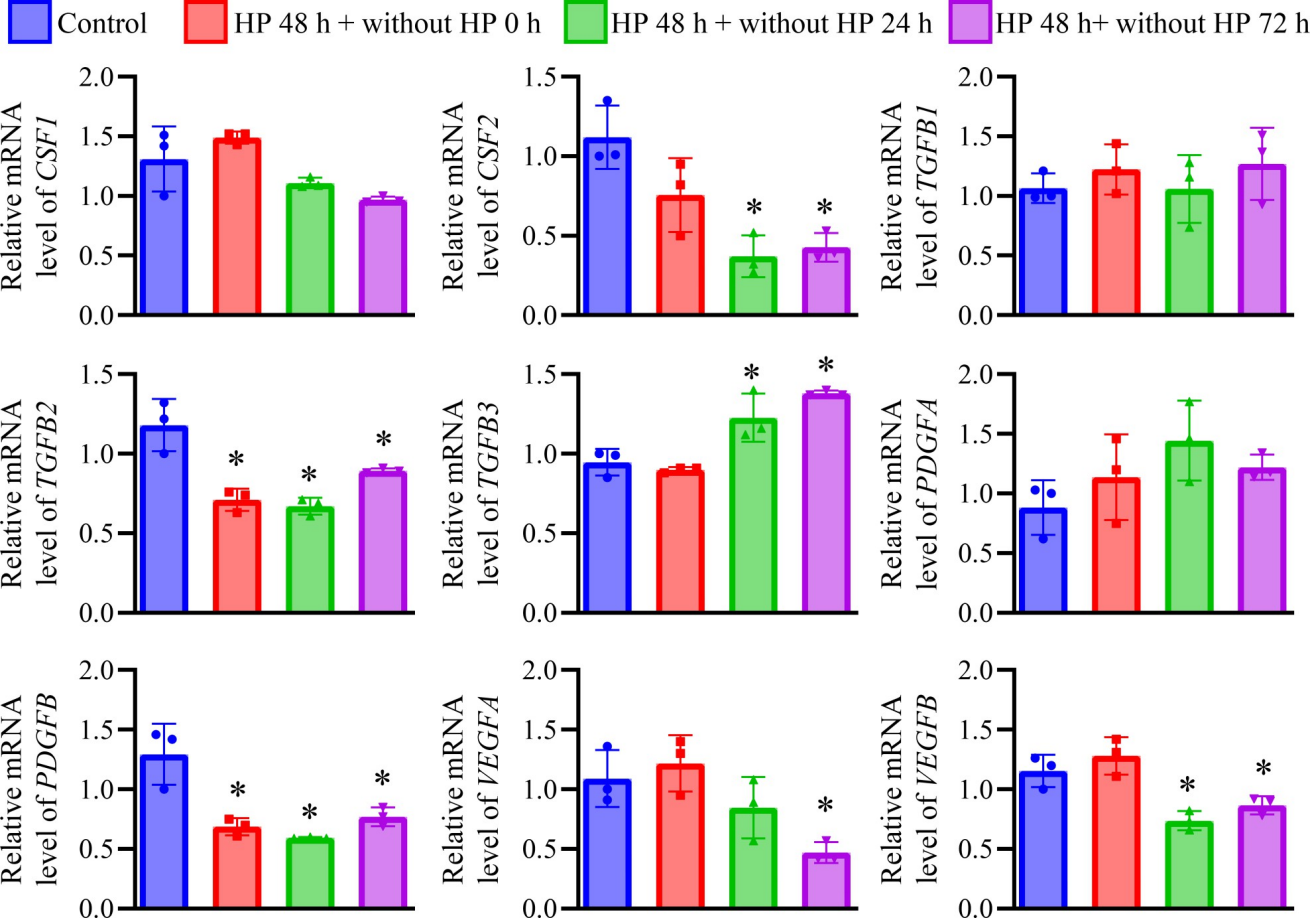
The expression of growth factors in HREpCs received 100 cmH_2_O HP treatment for 48 h. Quantitative analysis of the mRNA expression of *CSF1*, *CSF2*, *TGFB1*, *TGFB2*, *TGFB3*, *PDGFA*, *PDGFB*, *VEGFA*, and *VEGFB*. *: *p* < 0.05 *vs*. control cells.

We next investigated the expression of growth factors in HREpC after long-term HP treatment. The expression of *CSF2* and *VEGFB* was decreased in the HP-treated cells after removing from HP treatment (*p* < 0.05, *vs*. control, Fig 4). The expression of *TGFB2* and *PDGFB* was decreased after long-term HP treatment and subsequent removal from HP treatment (*p* < 0.05, *vs*. control, Fig 4). The expression of *TGFB3* was increased in the HP-treated cells after removing from HP treatment (*p* < 0.05, *vs*. control, Fig 4). The expression of *VEGFA* was decreased in the HP-treated cells 72 h after removing from HP treatment (*p* < 0.05, *vs*. control, Fig 4).

## Discussion

The physiological level of HP is known to regulate diverse cell fate and functions, including differentiation, migration, apoptosis, and proliferation [18]. However, intense and persistent changes in HP to abnormal levels can damage cells [19]. We have recently demonstrated that elevated HP induces profibrotic properties in hepatic stellate cells and atrial stromal cells [8, 20] and affects biphasically lymphocyte activation via complex mechanisms [21]. AKI is a frequent complication of obstructive uropathy, which confers a poor prognosis [1]. In human unobstructed kidneys, the IRPs range from zero to a few cmH_2_O, while in chronic kidney obstruction, they range from 68 to 95.2 cmH_2_O [2]. Therefore, we loaded HREpCs to 100 cmH2O HP to mimic an elevated HP condition by a pneumatic pressurizing system.

*Ex vivo* exposure of cells to an elevated HP condition has been widely studied [22, 23]. We found that HREpCs acquired an elongated morphology 48 h after 100 cmH_2_O HP treatment, but not 8 h after 100 cmH_2_O HP treatment, indicating long-term HP treatment may induce EMT in HREpCs. Moreover, the aspect ratio returns to the basic level 72 h after removing from HP treatment, suggesting elevated HP-induced elongation of HREpCs is reversible. In our previous study, we also found that the loading to 68 cmH_2_O HP for 24 h significantly changed the expression of EMT markers in HREpCs, such as vimentin, β-catenin, and α-SMA [12]. EMT of renal epithelial cells can be involved in interstitial fibrosis [24]. Therefore, biomechanical stresses, organ fibrogenesis, and EMT of tissue cells are likely linked to each other.

However, the effects of elevated HP-induced injury in renal epithelial cells remain unclear. It has been reported that growth factors regulate the pathogenesis of kidney disease and renal cell repair [25]. In a rat model, the expression of epidermal growth factor (*EGF*) and transforming growth factor-beta (*TGFB*) was increased in the post-obstructed kidney [26]. These authors suggest that *EGF* is involved in the promotion of recovery from obstructive uropathy. Meanwhile, *TGFB* is thought to play a major role in post-injured renal fibrosis, which might result in renal dysfunction [26]. Another study, using a mouse model, found that hepatocyte growth factor (*HGF*) attenuates apoptosis in renal cells and reduces the progression of interstitial fibrosis caused by obstructive uropathy [27]. In this study, our *ex vivo* data also showed that elevated HP disturbs the expression of growth factors in HREpCs, including *CSF2*, *TGFB*, platelet-derived growth factor (*PDGF)*, and vascular endothelial growth factor (*VEGF)*.

The role of *CSF2* in kidney disease has not been well investigated to date. One study has recently reported that *CSF2*-mediated macrophage transition promotes renal epithelial cell repair after kidney injury [28]. Our data showed that the expression of *CSF2* did not immediately change after the HP treatment. However, the expression of *CSF2* was decreased in the short-term HP-treated cells 72 h after removing from HP treatment. Similarly, the expression of *CSF2* persistently decreased in the long-term HP-treated cells 24 h after removing from HP treatment. Elevated HP induced a down-regulated expression of *CSF2*, which may impair renal cell repair.

*TGFB* is widely accepted as a critical mediator of renal fibrosis [29]. We have reported that ureteral obstruction increases the expression of *TGFB* in obstructed kidneys [14]. The *TGFB1*, *TGFB2*, and *TGFB3* isoforms are transcribed from different genes but have similar biological functions [29]. Our results showed that expression of *TGFB1* was increased after the short-term HP treatment, but returned to the basic level in 24h after removing from HP treatment. The expression of *TGFB2* was decreased by short-term HP treatment and returned to the basic level at 72h after removing from HP treatment. These results suggest that short-term HP-induced changes in *TGFB* expression are reversible. The expression of *TGFB3* was increased in the long-term HP-treated cells after removing from HP treatment. However, the expression of *TGFB2* was persistently decreased after loading to long-term HP. Meanwhile, *TGFB* also plays multiple physiological roles, such as cell proliferation, apoptosis, differentiation, autophagy, and immune responses [6]. Clinical studies of anti-TGFB therapies in kidney disease have failed due to a lack of beneficial effects on renal injury [30]. Thus, the role of *TGFB* in elevated HP-induced renal cell injury is conflicting and may depend on the type of *TGFB* isoform.

In the kidney, *PDGF* promotes the proliferation and recruitment of fibroblasts, mesangial cells, pericytes, and smooth muscle cells [31]. *PDGF* is critically involved in the progression of kidney diseases, including mesangioproliferative glomerulonephritis, interstitial fibrosis, acute kidney injury, and diabetic nephropathy [25, 31]. *PDGF* signaling is highly involved in fibroblast transformation, and capillary damage that results in alterations in renal hemodynamics [25]. We found that expression of *PDGF* was persistently decreased after loading to long-term HP. However, the role of *PDGF* in elevated HP-induced renal cell injury is unclear, and it may involve reciprocal interactions between other cells in the kidney.

*VEGF* stimulates glomerular endothelial cell and peri-tubular endothelial cell proliferation, migration, and survival [32]. It has been reported that *VEGF* is a double-edged sword in renal disease. Early *VEGF* supplementation protects against renal injury, whereas late anti-VEGF treatment attenuates renal fibrosis progression [33]. Moreover, *VEGF* inhibitors are commonly used in anti-angiogenic therapy of cancer, but these agents often cause renal dysfunction [34]. Higher serum VEGF levels are positively correlated with renal dysfunction in patients with diabetic nephropathy [35]. It has been suggested that the primary function of *VEGF* is to promote survival rather than angiogenesis [36]. In our study, we found that the expression of *VEGF* was increased after the short-term HP treatment, but returned to the basic level in 24h after removing from HP treatment. However, the expression of *VEGF* decreased in the long-term HP-treated cells after removing from HP treatment. These results suggest that the long-term HP treatment, but not short-term HP treatment, impaired *VEGF* expression and may result in renal dysfunction.

It has been reported that mechanical stress-related diseases are associated with various signaling pathways, such as TGFB/SMAD signaling pathway, RhoA/rho-associated protein kinase (ROCK) signaling pathway, and Wnt/β-catenin signaling pathway, etc. [37]. Pathological HP induces atrial fibroblast proliferation and collagen deposition through the TGFB/SMAD pathway [38]. Elevated HP activates the hepatic stellate cells to facilitate fibrosis progression via the cytoskeleton-related signals (i.e., RhoA, ROCK, and α-SMA) [8]. Research on the molecular mechanisms of elevated HP-induced kidney injury is lacking. Our previous study found that the loading to 68 cmH2O HP for 24 h significantly upregulated the expression of β-catenin in HREpCs, indicating the Wnt/β-catenin signaling pathway may be one of the major signaling pathways involved in HP-induced kidney injury [12].

This study has several limitations. Firstly, due to genetic and phenotypic changes in immortalized cells, we used primary HREpCs for the experiment. As these primary cells have very limited proliferative property, it is difficult to expand enough cells to isolate proteins for additional experiments to further confirm our findings from real-time PCR. Secondly, the potential morphological alterations of HP-treated HREpCs have not been extensively studied and remain largely unknown. Thus, we will further explore the changes in cell ultrastructure using transmission electron microscopy. Thirdly, it remains unclear how HP changes the expression of growth factors in renal epithelial cells. Therefore, it is critical to elucidate the relevant molecular mechanism associated with renal cell injury in response to elevated HP by further experiments. Lastly, obstructive uropathy-related AKI is caused not only by renal epithelial cells, but also by the reciprocal interactions between stromal cells, endothelial cells, and immune cells. Otherwise, it is critical to elucidate the key growth factors associated with obstructive uropathy-related AKI by further co-culture experiments and *in vivo* animal studies.

**Fig 5:**
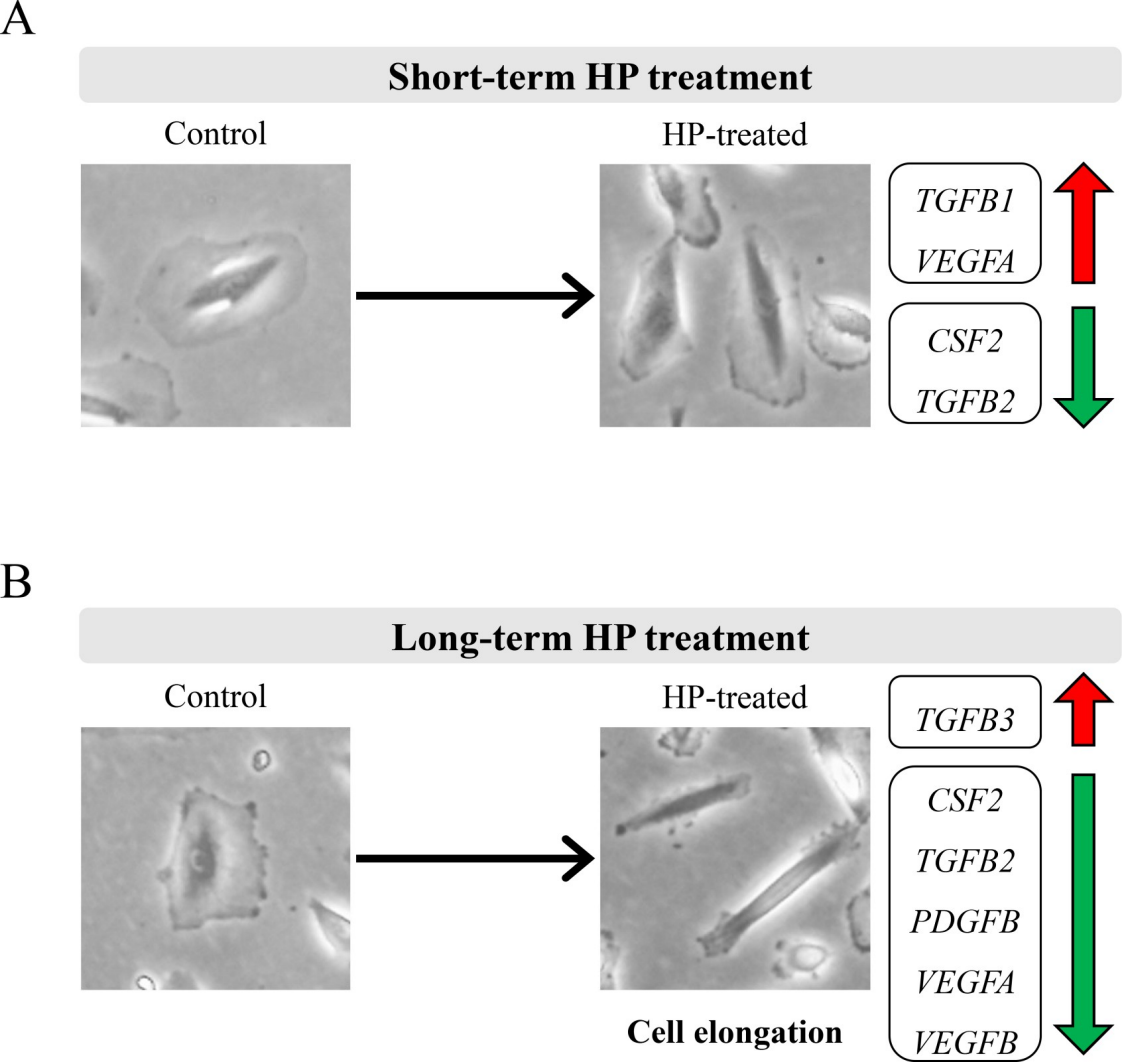
Graphical representation of the significant alterations due to HP treatment in HREpCs. (A) Short-term HP treatment induced mild changes in cell morphology and growth factor expression in HREpCs. (B) Long-term HP treatment induced severe changes in cell morphology and growth factor expression in HREpCs.

In conclusion, our *ex vivo* investigations suggest that long-term exposure of renal epithelial cells to elevated HP induces reversible morphology changes. Moreover, elevated HP disturbs the expression of growth factors in renal epithelial cells (Fig 5). Targeting key growth factors may provide a novel therapeutic approach to elevated HP-induced kidney injury, such as obstructive uropathy.

## Supporting information

S1 Table The list of primer sequences. (DOCX)

S1 File Raw data for figures. (XLSX)
