# Supplementary material for: Elevated hydrostatic pressure disturbs expression of growth factors in human renal epithelial cells
Source: PLoS One. 2024 Sep 6;19(9):e0310001. doi: 10.1371/journal.pone.0310001 (PMC11379293; doi:10.1371/journal.pone.0310001)
Supplement: S1 Table — (DOCX) [file pone.0310001.s001.docx]

**S1 Table. The list of primer sequences.**

| Gene name | Sense Primer | Anti-sense Primer |
| --- | --- | --- |
| GAPDH | GCAAATTCCATGGCACCGTCA | TTTTGGAGGGATCTCGCTCCTGG |
| CSF1 | GTTGCTGGAGAAGGTCAAGAATGTC | GCTTGGTCACCACATCTTGGCTG |
| CSF2 | GAAATGTTTGACCTCCAGGAGCCG | GAGGGCAGTGCTGCTTGTAGTG |
| TGFB1 | AATGGTGGAAACCCACAACGAAATC | CTTCTCGGAGCTCTGATGTGTTGAA |
| TGFB2 | GAAGAGTACTACGCCAAGGAGGTTT | TGTAACAACTGGGCAGACAGTTTCG |
| TGFB3 | GTGAGTGGCTGTTGAGAAGAGAGTC | ATCCTCATTGTCCACGCCTTTGAAT |
| PDGFA | ATGTTCTGGCCGAGGAAGCC | GGAGTCTATCTCCAGGAGTCGC |
| PDGFB | TTGATGATCTCCAACGCCTGCT | AATGGTCAGGGAACCCAGGCT |
| VEGFA | GCACAACAAATGTGAATGCAGACCAA | CGCTCCAGGACTTATACCGGGATTT |
| VEGFB | CCAAGTCCGGATGCAGATCCTC | CATTCACACTGGCTGTGTTCTTCCA |
